# Rapid identification and subsequent contextualization of an outbreak of methicillin-resistant Staphylococcus aureus in a neonatal intensive care unit using nanopore sequencing

**DOI:** 10.1099/mgen.0.001273

**Published:** 2024-07-05

**Authors:** Rhys T. White, Sarah Bakker, Megan Burton, M. Leticia Castro, Christine Couldrey, Kristin Dyet, Alexandra Eustace, Chad Harland, Samantha Hutton, Donia Macartney-Coxson, Claire Tarring, Charles Velasco, Emma M. Voss, John Williamson, Max Bloomfield

**Affiliations:** 1 Institute of Environmental Science and Research, Health Group, Porirua 5022, New Zealand; 2 Awanui Labs Wellington, Department of Microbiology and Molecular Pathology, Wellington 6021, New Zealand; 3 Livestock Improvement Corporation, Research and Development, Newstead 3286, New Zealand; 4 University of Otago, Department of Microbiology and Immunology, Dunedin 9016, New Zealand; 5 Te Whatu Ora/Health New Zealand, Infection Prevention and Control, Capital, Coast & Hutt Valley, Wellington 6021, New Zealand

**Keywords:** antibiotic resistance, genomic surveillance, infection control, outbreak detection, phylogenetic analysis

## Abstract

Outbreaks of methicillin-resistant *Staphylococcus aureus* (MRSA) are well described in the neonatal intensive care unit (NICU) setting. Genomics has revolutionized the investigation of such outbreaks; however, to date, this has largely been completed retrospectively and has typically relied on short-read platforms. In 2022, our laboratory established a prospective genomic surveillance system using Oxford Nanopore Technologies sequencing for rapid outbreak detection. Herein, using this system, we describe the detection and control of an outbreak of sequence-type (ST)97 MRSA in our NICU. The outbreak was identified 13 days after the first MRSA-positive culture and at a point where there were only two known cases. Ward screening rapidly defined the extent of the outbreak, with six other infants found to be colonized. There was minimal transmission once the outbreak had been detected and appropriate infection control measures had been instituted; only two further ST97 cases were detected, along with three unrelated non-ST97 MRSA cases. To contextualize the outbreak, core-genome single-nucleotide variants were identified for phylogenetic analysis after *de novo* assembly of nanopore data. Comparisons with global (*n*=45) and national surveillance (*n*=35) ST97 genomes revealed the stepwise evolution of methicillin resistance within this ST97 subset. A distinct cluster comprising nine of the ten ST97-IVa genomes from the NICU was identified, with strains from 2020 to 2022 national surveillance serving as outgroups to this cluster. One ST97-IVa genome presumed to be part of the outbreak formed an outgroup and was retrospectively excluded. A second phylogeny was created using Illumina sequencing, which considerably reduced the branch lengths of the NICU isolates on the phylogenetic tree. However, the overall tree topology and conclusions were unchanged, with the exception of the NICU outbreak cluster, where differences in branch lengths were observed. This analysis demonstrated the ability of a nanopore-only prospective genomic surveillance system to rapidly identify and contextualize an outbreak of MRSA in a NICU.

**Impact Statement**

This research emphasizes the significance of utilizing genomics for prospective monitoring to promptly identify methicillin-resistant *Staphylococcus aureus* (MRSA) outbreaks in a neonatal intensive care unit. An MRSA outbreak was detected only 13 days from the initial positive sample being collected, enabling prompt implementation of infection prevention and control measures, thus limiting potential negative impacts on the infants on the unit and the functioning of the unit as a whole. In addition, the research showed diverse lineage categorizations, underscoring the importance of genomics in comprehending antibiotic resistance evolution. The results show that nanopore sequencing is a reliable platform for studying outbreaks because it is cost-effective and provides quick results. In addition to healthcare, the study also points out the relationship between human and animal sources of *S. aureus*, which could have implications for veterinary medicine and agricultural systems. The study enhances our understanding of antibiotic resistance development and dissemination by examining the evolutionary history of MRSA strains.

## Data Summary

The study sequences are available in the National Center for Biotechnology Information (NCBI) under BioProject accession number PRJNA1046639. The raw sequence read data generated in this study have been deposited to the NCBI sequence read archive (SRA, https://www.ncbi.nlm.nih.gov/sra) under accession numbers SRR26992863 to SRR26992891. The complete assembly for strain 23MR1425 has been deposited to GenBank under accession numbers CP143800 and CP143801. The software used to analyse raw sequence reads for polymorphism discovery and whole-genome sequencing-based phylogenetic reconstruction are available as described in the Methods. The authors confirm that all supporting data protocols have been provided in the article or supplementary data files.

## Introduction

*Staphylococcus aureus* is a Gram-positive pathogen that can lead to severe opportunistic infections in clinical settings [1]. Despite advancements in combating such pathogens [2,3], the emergence of antibiotic-resistant strains, particularly methicillin-resistant *S. aureus* (MRSA), has posed formidable challenges globally [4,8]. MRSA and methicillin-susceptible *S. aureus* (MSSA) are common causes of hospital-associated and community-associated infections [4,8].

MRSA outbreaks in healthcare settings, especially in neonatal intensive care units (NICUs), have become a well-recognized global concern [9]. Neonates, especially preterm and low-birthweight infants, face higher risks of MRSA colonization and infection due to their underdeveloped protective microbiota and immature immune systems [10]. This increased vulnerability is exacerbated by evidence of nosocomial and neonatal transmission of *S. aureus* involving environmental reservoirs as well as colonized parents and healthcare workers [9].

Such outbreaks threaten the ability to safely deliver healthcare to this vulnerable population and are highly disruptive to models of care, especially if prolonged [11,13]. Genomics has improved the understanding of the transmission dynamics of MRSA in NICU settings and has been used to identify and control outbreaks [13,15]. This has been in the form of retrospective or reactive investigations instigated when outbreaks have reached sufficient size to be detected based on standard surveillance [13,14]. To our knowledge, the utility of prospective/proactive genomic surveillance in this setting is less well described, as is the use of Oxford Nanopore Technologies (ONT) sequencing as a standalone sequencing platform [16].

In 2022 we instituted a prospective genomic surveillance programme using ONT sequencing to target several common hospital pathogens, one of which was MRSA isolated from patients in the NICU of our institution [17]. The programme aimed to detect potential outbreaks early to enable effective infection prevention and control (IPC) interventions and limit the negative impacts of such outbreaks [18]. Here, we describe the rapid detection and control of an outbreak of sequence type (ST)97 MRSA in our NICU using this surveillance programme. As a proof-of-concept, we explored an alternative approach to the conventional Illumina sequencing used in New Zealand’s genomic surveillance landscape [19]. By integrating nanopore sequencing, our study offers a fresh perspective in the broader phylogeny and evolution of ST97 MRSA.

Additionally, *S. aureus* ST97 is a predominant pathogen in bovine cattle worldwide [20]. Given New Zealand’s isolated environment and heavy dependence on agriculture as a fundamental pillar of its economy and export industry [21], it is crucial to understand whether there are clear genomic differences between ST97 strains isolated from humans and bovine hosts. Studies on MRSA in New Zealand cattle are limited, with only preliminary data available [22,23]. By comparing agricultural samples, our research investigates potential zoonotic links, emphasizing the importance of a One Health approach in managing MRSA across different reservoirs. This shift underscores the importance of adopting advanced genomic technologies to enhance our understanding and control of infectious diseases in healthcare settings.

## Methods

### Setting

New Zealand (known as Aotearoa in the Māori language) is an island nation in the Southwest Pacific, with a population of around 5.27 million. Wellington Regional Hospital (WRH) provides tertiary services to the lower North Island/Te Ika-a-Māui of New Zealand, serving a population of around 500 000. The WRH NICU is a Level 3 unit resourced for 37 infants. It provides advanced care for extremely premature infants (born as early as 24 weeks of gestation) and for infants in need of ventilation, intravenous feeding, and other forms of intensive care monitoring and treatment. Routine MRSA screening is not performed.

Awanui Laboratories Wellington, formerly Southern Community Laboratories (SCL), is a medium-sized laboratory that provides clinical diagnostic services to WRH and the local region. The microbiology and molecular departments process around 300 000 samples yearly. The analysis and reporting of this outbreak constituted an ‘audit or related activity’ as per New Zealand Health and Disability Ethics Committees, so it did not require review.

### Genomic surveillance programme

From the beginning of 2022, all MRSA isolated from infants in the unit were prospectively sequenced at Awanui Laboratories [17]. Isolates for sequencing are batched and run on a weekly or fortnightly basis. Details describing sampling and extraction of DNA can be found in the Supplementary Materials. Methicillin resistance was determined phenotypically for all isolates using the Vitek II instrument (bioMerieux) and the AST-P656 card, according to European Committee on Antimicrobial Susceptibility Testing (EUCAST) methods [24].

For isolates sequenced before March 2023 (sa220609barcode87 and sa230215barcode55), libraries were constructed using 50 ng of genomic DNA with the ONT rapid barcoding kit 96 (SQK-RBK110-96) as per the manufacturer’s instructions. Subsequently, the entire library was loaded onto an R9.4 flow cell (FLO-MIN106) and run on a MinION device for approximately 20–40 h (using MinKNOW v22.10.10). After March 2023 (i.e. including when the outbreak occurred), sequencing used Q20+ chemistry; libraries were created using 50–100 ng of genomic DNA, prepared using the rapid barcoding kit 96 (SQK-RBK114-96) and sequenced on an R10.4.1 flow cell (FLO-MIN114) with MinKNOW v23.04.5. Krocus v1.0.3 [25] was used to generate a rapid multi-locus sequence type (MLST) from the raw FASTQ files. After each sequencing run, MLSTs were compared to other recent MRSA isolates in the NICU. Awanui Laboratories does not have next-generation sequencing bioinformatics expertise, so if greater than expected numbers of a given MLST were observed, further investigations were instigated by the IPC team and data were transferred to the Institute of Environmental Science and Research (ESR) for more granular analysis.

### Retrospective genome analyses

Original Fast5 sequence files were converted to Pod5 using pod5 v0.3.2 (https://github.com/nanoporetech/pod5-file-format, accessed on 18 March 2024) and then basecalled using Dorado v0.3.4 (https://github.com/nanoporetech/dorado, accessed on 18 March 2024). The base-calling process was carried out using the ‘super accuracy’ models, with a batch size of 2008 and a chunk size of 1000, while parameters were kept at their default settings. Further details of laboratory methods including: nanopore read quality control, Illumina sequencing, genome assembly, multilocus sequence typing, virulence and antibiotic resistance gene genotyping, and public data curation, are available in the Supplementary Materials.

### Genome annotation for 23MR1425

In the absence of an available ST97 reference genome representing New Zealand *S. aureus* strains, we selected 23MR1425, a clinical isolate collected from a neonatal eye swab, as our reference genome (index case on the WRH NICU identified in June 2023). The assembly representing strain 23MR1425 was annotated using Prokka v1.14.6 [26]. Prophage regions were identified using PHASTER [27,28] and then annotated using Pharokka v1.5.1 [29]. Mobile genetic elements were identified using IslandViewer 4 [30] and ISsaga v2.0 [31] (ISfinder platform [32]), followed by manual curation using Artemis v18.2.0 [33].

### Assembly-based variant detection and ST97 phylogenetic analyses

Further details of public data curation and *de novo* assembly are available in the Supplementary Materials. A core-genome alignment was generated from 679 *S*. *aureus* ST97 genomes using Parsnp v1.7.4 [34] with the 23MR1425 chromosome serving as the reference to call single-nucleotide variants (SNVs) (index ST97 MRSA case, date sample received June 2023). Resulting SNV alignments were used to reconstruct phylogenies. Maximum-likelihood phylogenetic trees were reconstructed using RaxML v8.2.12 [35] (GTR-GAMMA correction) by optimizing 20 distinct, randomized maximum-parsimony trees before adding 1000 bootstrap replicates. The resulting phylogenetic trees were visualized using FigTree v1.4.4 (http://tree.bio.ed.ac.uk/software/figtree/, accessed on 18 March 2024). To further identify robust phylogenetic groups within the ST97 phylogeny, we used the core-genome SNV alignment as input into rhierBAPS v1.0.1 [36] [an R v4.3.2 [37] implementation of hierarchical Bayesian Analysis of Population Structure (BAPS) [38]] with one level of clustering, allowing up to ten initial clusters.

### High-resolution cluster phylogeny

High-resolution analyses of genetic variants were performed using Burrows-Wheeler Aligner (BWA) v0.7.17 [39]; BEDTools v2.28.0 [40]; seqtk v1.3-r106 (https://github.com/lh3/seqtk, accessed on 15 September 2023); Trimmomatic v0.36 [41]; pindel [42]; Mosdepth [43]; SAMtools v1.9 [44]; Picard v2.7.1 (https://github.com/broadinstitute/picard, accessed on 18 March 2024); the Genome Analysis Tool Kit v4.3.0.0 (GATK) [45,46]; and SNPEff v4.3.1t [47], as implemented in SPANDx v4.0 [48]. Resulting SNV alignments were used to reconstruct phylogenies. The pairwise SNV distances were determined using snp-dist v0.6.3 (https://github.com/tseemann/snp-dists, accessed on 18 March 2024). Maximum-parsimony trees were reconstructed using the heuristic search feature of PAUP v4.0a [49]. The resulting phylogenetic trees were visualized using FigTree v1.4.4. Notably, this analysis defines a core genome as regions estimated to the nearest 100 bp with ≥95 % coverage across one or more genomes in the given population.

### Divergence estimates of a subset of Clade 1.1 *S. aureus* ST97

To calibrate the phylogeny, we used tip-dating approaches using TempEst v1.5.3 [50] and BEAST2 v2.7.1 [51,52]. For this analysis, we utilized Illumina sequence data due to their higher accuracy and lower error rates compared to Nanopore sequencing. This choice ensures robust calibration of the phylogeny and improves the reliability of evolutionary timeline estimations. For the Bayesian approach, we first determined whether the strict or optimized relaxed clock (with a log-normal distributed rate) model best fits our dataset. Using the tip date’s function, six models representative of a strict clock model and an optimized relaxed log-normal clock model were set up. The Bayesian skyline, coalescent constant and exponential growth population size change models were compared for each clock model to ensure the selection of the best-fit model. The Gamma Site Model Category Count was set to four, and the GTR substitution model rates determined from jModelTest v2.1.10 [53] were included (i.e. rate AC=0.93, AG=3.20, AT=0.83, CG=0.21, CT=3.39 and GT=1.00). The initial clock rate was set to 5.67×10^−4^ substitutions per site per year (estimated from the root-to-tip regression analysis in TempEst) with a uniform distribution and an upper bound of 0.1. All other priors were left as default. All models were tested with the Nested Sampling Bayesian computation algorithm v1.2.1 within the BEAST2 package with a particle count of 32, sub-chain length of 5000 and Epsilon of 1.0×10^−12^.

Once the best-fitting tree model was determined, three independent Markov chain Monte Carlo generations were conducted for 100 million generations for each analysis. Trees were sampled every 1000 generations, resulting in triplicate samples of 100 000 trees for each model test. To assess statistics, all BEAST2 runs were imported into Tracer v1.7.2 (http://github.com/beast-dev/tracer/, accessed on 18 March 2024). LogCombiner v2.7.1 (BEAST2 package) then combined the replicated analyses for each model with a 10 % burn-in to assess convergence. Finally, TreeAnnotator v2.7.1 (BEAST2 package) removed the 10 % burn-in and generated maximum clade credibility trees for each run (established from 243 million trees), reporting median values with a posterior probability limit set at 0.5. The resulting phylogenetic trees were visualized using FigTree v1.4.4.

## Results

### Outbreak detection

**Fig. 1.**
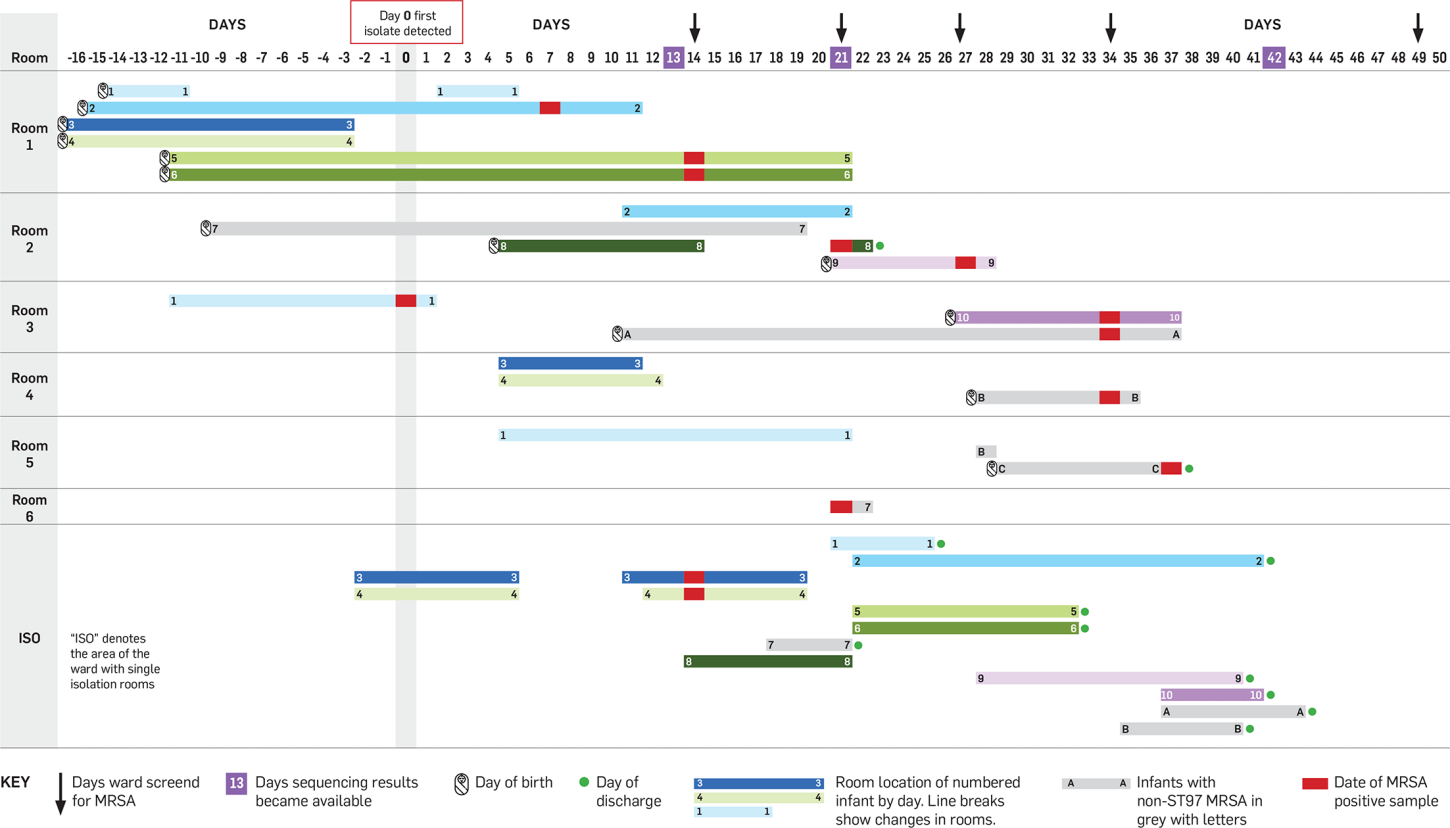
Line chart of neonatal intensive care unit MRSA ST97 outbreak. Days are displayed on the *x*-axis, with the day the first isolate was collected denoted as day 0 (actual dates have been omitted for patient privacy). Each horizontal shaded bar represents the days spent by the numbered infant in the room as indicated on the *y*-axis. Infants with non-ST97 MRSA are shown in lighter shaded bars and denoted with letters instead of numbers. Infant 7 was ST97 but was excluded from the outbreak based on the phylogenetic analysis, so is also shown in light grey.

During a routine weekly sequencing run in June 2023, two NICU MRSA isolates recovered from eye swabs were identified as ST97. This was an uncommon ST for the NICU, and the infants had spent time in the same room, so a possible transmission event was suspected (Fig. 1). The following day, all infants who had been cared for in the same rooms as the two index cases were screened for MRSA (combined nasal, axilla, umbilicus and perineum swabs), which detected a further four infants colonized with MRSA (two sets of twins; infants 3–4 and 5–6, Fig. 1). These infants were immediately placed in contact isolation. The following weekly sequencing run was brought forward, and these four isolates were also shown to be ST97. At this point, all infants in the NICU were screened for MRSA, and this detected two more cases. Three additional rounds of ward screening were undertaken over 4 weeks, detecting a further two cases of ST97 MRSA, after which the outbreak was declared closed. Screening also revealed three infants with non-ST97 MRSA (infants A–C, Fig. 1), who could be rapidly excluded from the outbreak. The outbreak totalled ten infants, none of whom developed invasive infection with MRSA (Table S1, available in the online version of this article). The outbreak was identified 13 days after the collection of the initial MRSA-positive sample from infant 1 and closed 38 days later.

### A high-quality reference genome was generated for the index *S. aureus* ST97 case

We performed initial nanopore whole-genome sequencing (WGS) on 23MR1425, which yielded 102 227 single-ended reads, providing complete genome coverage at an average depth of 133×. The median read length was 3 160 bp (N50=10 546 bp), and the median read quality score was 15.4. The *de novo* nanopore assembly, further refined with Illumina reads, revealed that 23MR1425 possesses a circular chromosome with a length of 2 753 159 bp and an average GC content of 32.86 % (Fig. S1a). *In silico* analysis determined that 23MR1425 is *spa* type t359. Additionally, the chromosome of 23MR1425 was predicted to harbour a type IVa(2B) staphylococcal cassette chromosome *mec* (SCC*mec*) which carried the *mecA* gene (encoding penicillin-binding protein 2a). We comprehensively characterized the chromosomal attributes of 23MR1425 through BLASTn comparison with 18 other complete *S. aureus* genomes downloaded from GenBank (Fig. S2). Notably, the chromosome of 23MR1425 contained a conserved prophage, designated 23MR1425_prophage1, which was also found in six other publicly available ST97 genomes. In contrast, 23MR1425_prophage2, housing the *sak* gene responsible for encoding staphylokinase (protease III), a virulence factor involved in clot dissolution, appeared to be absent from the ST97 chromosome of MOK063 (GenBank: CP029629). Remarkably, the chromosome of 23MR1425 represents the first complete ST97 chromosome displaying the integration of the type IVa(2B) SCC*mec* element. The complete genome of 23MR1425 included the plasmid p23MR1425A (20 403 bp, rep20 plasmid), which houses a *bla* operon putatively responsible for penicillin resistance. This *bla* operon is positioned downstream of an IS*Sau6* insertion sequence element on p23MR1425A (Fig. S1b). A concise overview of genes encoding antibiotic resistance and virulence factors in the chromosome or plasmid of 23MR1425 is shown in Table S2.

### Genomic diversity and lineage classification of *S. aureus* ST97 reveals two primary clades with distinct origins

In this study, we analysed 679 genomes in total. These included: six complete genomes from NCBI, 468 draft genomes from the PathogenWatch platform (https://pathogen.watch/, accessed on 25 August 2023), 127 bovine genomes from Livestock Improvement Corporation (LIC) trials and 13 human genomes sourced from the Pathlab (Supplementary Materials), 35 from ESR national staphylococcal surveillance surveys, 18 draft genomes from bovine mastitis-causing *S. aureus* in New Zealand [54] and 12 ST97 genomes from WRH NICU. The nanopore sequence read data quality metrics (Table S3) and *de novo* assembly quality metrics (Table S4) for the 12 ST97 genomes from WRH are presented in the Supplementary Materials. Additionally, the assembly metrics for the 492 publicly available genomes are outlined in Table S5.

**Fig. 2.**
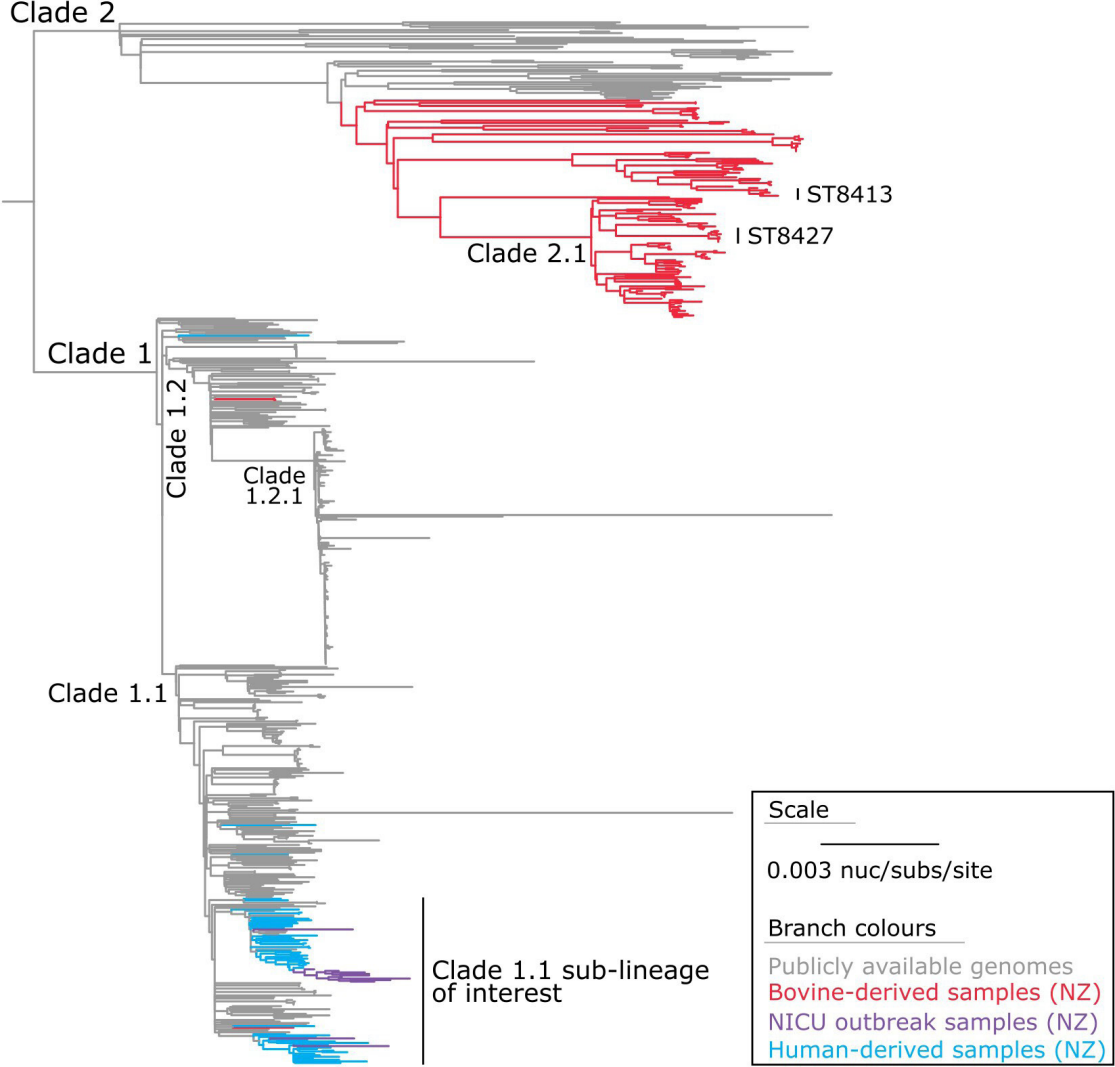
Maximum-likelihood phylogeny of *S. aureus* ST97. The phylogeny was inferred from 28 634 core-genome single-nucleotide variants (SNVs) from 679 assembled genomes. SNVs were derived from a core-genome alignment of 2 087 573 bp and are called against the chromosome of sample 23MR1425. The phylogenetic tree is rooted at the midpoint, which corresponds to the actual root by *S. aureus* ST834 strain 70017 (SRA accession: DRR291698), which has been omitted for visualization.

When using nanopore-only data (for the NICU outbreak genome), we identified 28 634 core-genome SNVs from the 679 genomes (Fig. 2). These SNVs were called against the chromosome of sample 23MR1425 using assembly-based variant detection (see Methods). The phylogenetic tree is rooted at the midpoint (Fig. 2), which corresponds to the actual root by *S. aureus* ST834 strain 70017 (SRA accession: DRR291698) (Fig. S3).

Considering our findings, *S. aureus* ST97 can be broadly classified into two primary lineages: Clade 1, predominantly consisting of genomes sourced from humans, and Clade 2, primarily consisting of genomes from bovine origins. Notably, within Clade 1, we observed two bovine-derived genomes, B791 and B792, which cluster within sub-clade 1.2, and another genome, B371 (collected from a non-aseptic bulk milk tank in the North Island/Te Ika-a-Māui in 2021), which clusters in sub-clade 1.1. Of particular interest, a substantial portion of the *S. aureus* genomes obtained through ESR national surveillance surveys (*n*=35) and a separate group of genomes from the Pathlab (*n*=10) and a genome from an LIC trial (*n*=1) coalesce within a sub-lineage of sub-clade 1.1, alongside the NICU outbreak genomes (*n*=12) (Fig. 2).

### Genomic comparison of *S. aureus* ST97 sub-clade 1.1 reveals consistency between nanopore and Illumina sequencing

**Fig. 3.**
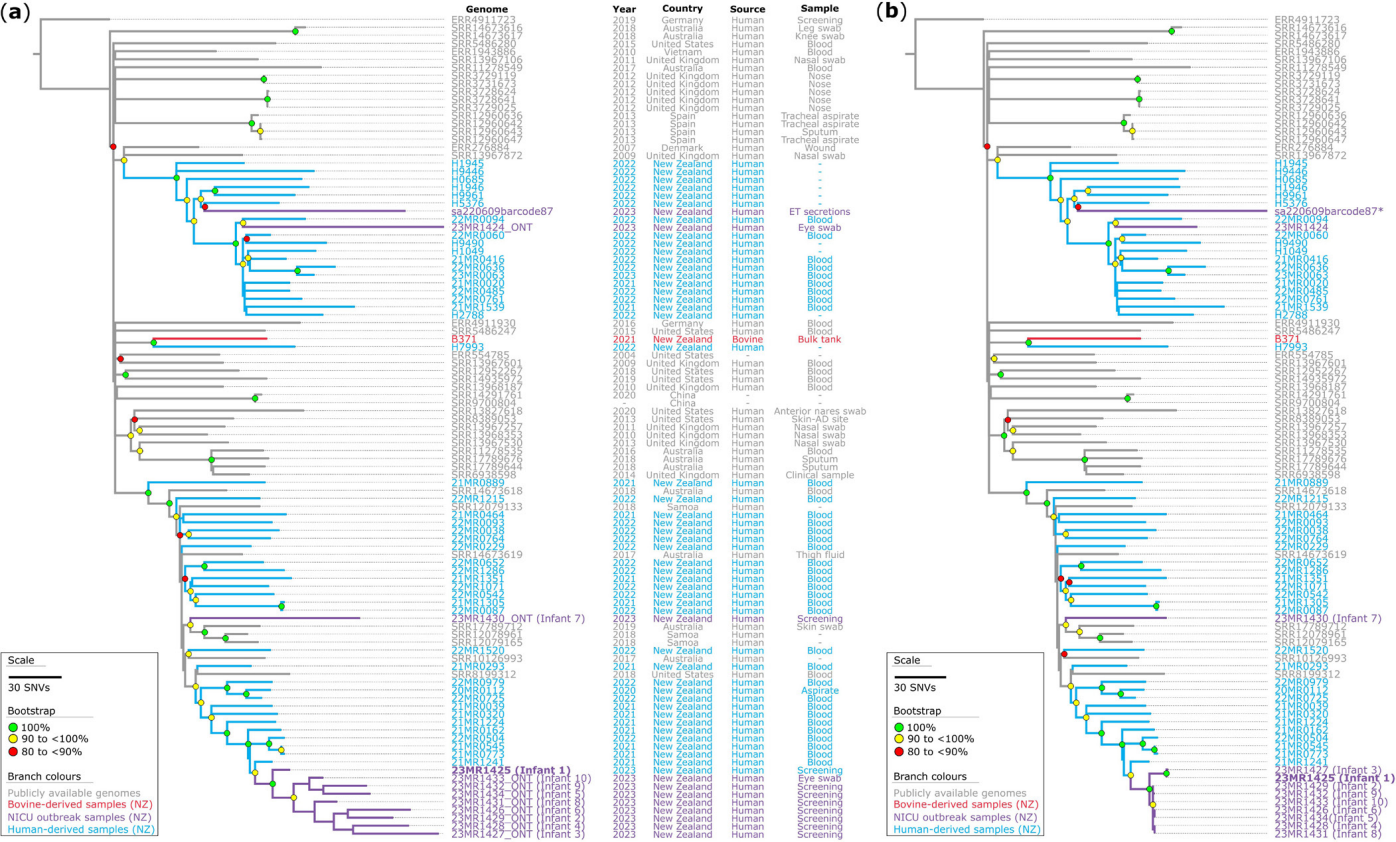
Maximum-parsimony phylogeny of a subset of Clade 1.1 *S. aureus* ST97 isolates. (**a**) The phylogeny is based on nanopore data for the neonatal intensive care unit (NICU) isolates (denoted by ONT in taxon labels). The phylogeny was inferred from 4797 core-genome single-nucleotide variants (SNVs) from 103 genomes. SNVs were derived from a core-genome alignment of ~2 560 000 bp and were called against the chromosome of sample 23MR1425. The consistency index for the tree was 0.96. (**b**) The phylogeny is based on Illumina data for the NICU isolates. The phylogeny was inferred from 4651 core-genome SNVs from 103 genomes. SNVs were derived from a core-genome alignment of ~2 602 000 bp and are called against the chromosome of sample 23MR1425. The genome for sample sa220609barcode87 (*) represents nanopore-only sequence data. The consistency index for the tree is 0.99. SNV density filtering in SPANDx (excluded regions with three or more SNVs in a 10 bp window). Both phylogenetic trees were rooted according to the ERR4911723 outgroup. Bootstrap values >80 % (1000 replicates) are shown.

Utilizing the assembly-based ST97 phylogeny above, we could narrow our analysis to the genomic data of strains within the specific sub-clade 1.1 sub-lineage of interest (Fig. 2). This analysis assessed the genomic diversity of 12 high-quality *S. aureus* genomes from neonatal hosts in New Zealand, comparing them with 45 publicly available draft *S. aureus* ST97 Clade 1.1 sub-lineage genomes (Fig. 3). The analysis also included 35 ST97 genomes from ESR national staphylococcal surveillance surveys, ten genomes from the Pathlab and one LIC trial isolate. The initial objective was to compare the genomes sequenced using nanopore sequencing to determine if the genomes could be grouped together using nanopore-only sequencing data without Illumina data and achieve comparable results (Fig. 3a). To do this, we generated a maximum-parsimony phylogenetic tree based on 4797 core-genome SNVs from 103 genomes, using 23MR1425 as the reference genome (see Methods). We then repeated the experiment using the same genomic data; however, we replaced the nanopore-only assemblies from 12 ST97 genomes with Illumina sequence read data. We generated another maximum-parsimony phylogenetic tree based on 4653 core-genome SNVs from 103 genomes (Fig. 3b). These phylogenetic analyses revealed notable differences with different sequencing technologies. In both phylogenetic trees, we observed robustly supported branches, reaffirming the accuracy of our evolutionary conclusions. Notably, one sample, sa220609barcode87, exclusively relied on nanopore sequencing due to the unavailability of an isolate for Illumina sequencing (collected in June 2022).

The sa220609barcode87 and 23MR1424 genomes were methicillin-susceptible and clustered with 18 other MSSA ST97 Clade 1.1 sub-lineage genomes from New Zealand. On the other hand, the ten MRSA ST97 genomes collected from the NICU clustered with 27 other MRSA genomes collected from New Zealand as part of the ESR surveillance. The genome of 23MR1430/infant 7 was ST97 and thought to be part of the outbreak; however, it was shown to be an outgroup to the other ST97 isolates, so was retrospectively excluded from the outbreak (Fig. 3a and b). Both phylogenetic trees were nearly identical, except for the NICU outbreak cluster (Fig. 3a and b). The nanopore-only genomes exhibited elongated and potentially spurious branches in the phylogenetic tree (Fig. 3a), with a median pairwise SNV distance of 65 [interquartile range (IQR): 51–75; range: 37–94] observed within the main outbreak cluster. In contrast, the Illumina genomes yielded shorter branches (Fig. 3b), and a median pairwise SNV distance of 2 (IQR: 1–13; range: 0–14), which is within previously proposed cutoffs [55]. This difference is particularly notable for isolates 23MR1425/infant 1 and 23MR1427/infant 3, where the Illumina data indicate they may share ten additional SNVs, separating them from the main outbreak cluster.

It is worth noting that the genomes for B371 (collected in 2021 from a bovine non-aseptic bulk milk tank in the North Island/Te Ika-a-Māui) and H7993 (collected in 2022 from a human sample in the North Island/Te Ika-a-Māui) clustered together, albeit with a pairwise SNV distance, determined using snp-dist v0.6.3 (https://github.com/tseemann/snp-dists, accessed on 31 May 2024), of 148 core-genome SNVs separating them (Fig. 3).

### Evolutionary timeline of a subset of Clade 1.1 ST97 from New Zealand

The evolutionary timeline of a subset of Clade 1.1 ST97 from New Zealand was examined in this study. First, we assessed the temporal signal of the dataset by constructing a maximum-likelihood phylogeny in TempEst (Fig. S4). The analysis revealed that the ST97 genomes (*n*=97) exhibited a linear relationship (correlation coefficient=0.72) between divergence time and evolutionary distance. Regression analysis in TempEst indicated that these genomes accumulated mutations at a rate of 5.67×10^−4^ substitutions per site per year (*R*^2^=0.52) (Fig. S4b). This analysis, while exploratory, suggested that this dataset exhibits a clock-like behaviour. According to the root-to-tip divergence analysis, the most recent common ancestor (MRCA) of this subset of Clade 1.1 ST97 lineage was estimated to have emerged in 1979 (95 % confidence interval: 1968–1988). Additionally, an SNV density plot (Fig. S5) showing the number of SNVs across a 1 000 bp sliding window did not reveal any regions in the core-genome SNV alignment with >15 SNVs in a 1 000 bp window relative to the reference genome (23MR1425), supporting the absence of any substantial recombination in this particular phylogenetic analysis. This was later confirmed with additional recombination filtering (Supplementary Materials), which identified seven putative recombinogenic regions (relative to the reference genome 2314MR25) containing only five of the total 4189 core-genome SNVs (Table S6). Excluding these regions had minimal effects on the phylogeny (Fig. S6), and therefore it was decided not to exclude these five SNVs in downstream analyses [56].

**Fig. 4.**
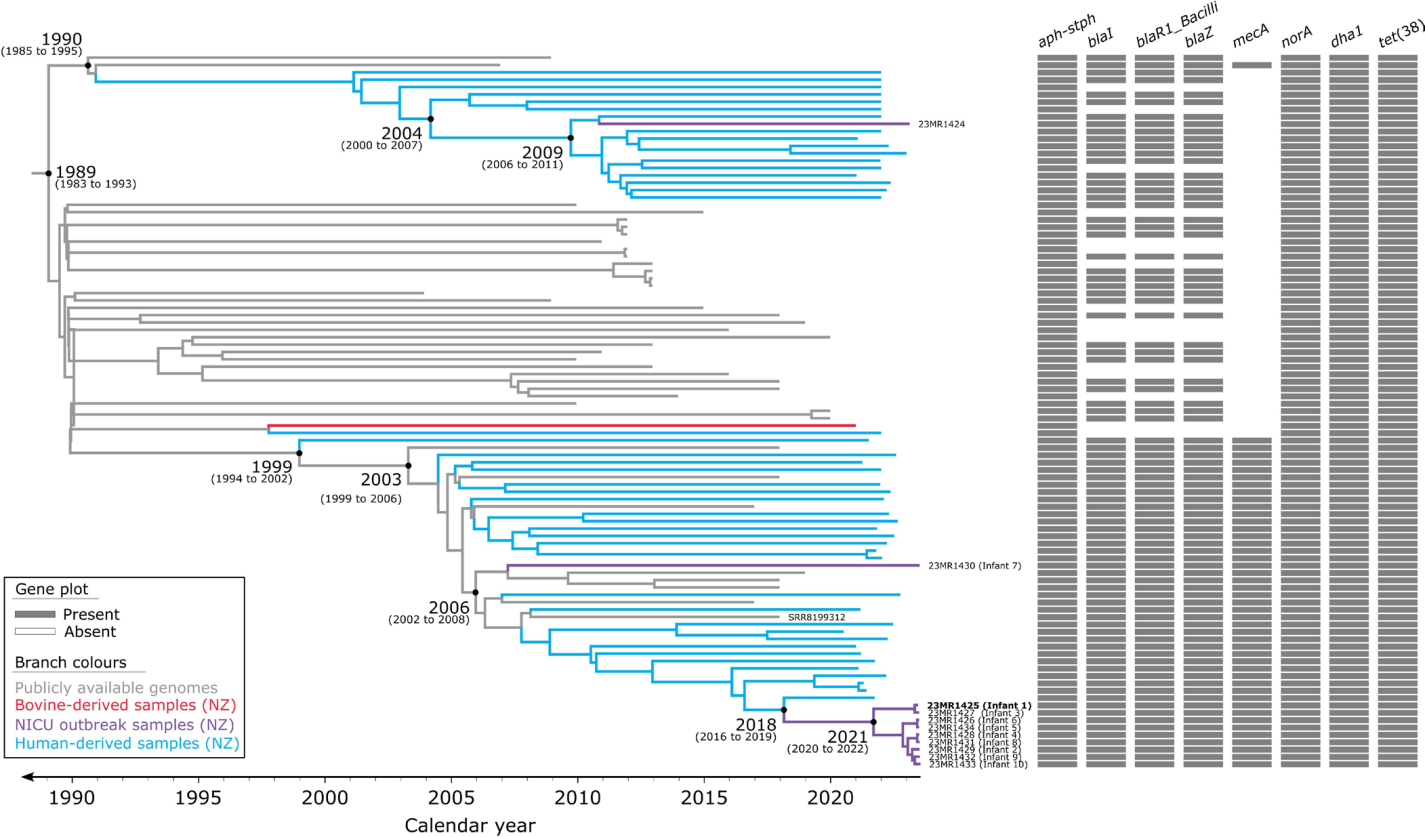
Evolutionary reconstruction of a subset of Clade 1.1 *S. aureus* ST97 isolates. A time-calibrated maximum clade credibility tree was inferred from 4189 core-genome single-nucleotide variants (SNVs) from 97 ST97 genomes. SNVs were derived from a core-genome alignment of ~2 605 600 bp and were called against the chromosome of sample 23MR1425 (bold). SNV density filtering in SPANDx (excluded regions with three or more SNVs in a 10 bp window). The *x*-axis represents the emergence time estimates.

Once we confirmed the presence of a temporal signal in the ST97 dataset (*n*=97), we employed the Nested Sampling Bayesian computation algorithm to determine the best-fitting tree model and generate a time-calibrated phylogeny. The results of the Nested Sampling algorithm favoured the combination of a strict clock model and the Bayesian skyline population size change model, with a marginal likelihood estimate of −33 211.28 (sd: ±4.14) (Table S7). A BEAST2-based phylogenetic analysis revealed fine-detail relationships among the subset of ST97 strains, leading to the identification of two major sub-lineages. The MRCA of these two major sub-lineages was estimated to have emerged around 1989 [95 % highest posterior density (HPD): 1983–1993] (Fig. 4). The median evolutionary rate determined by BEAST2 is 8.18×10^−4^ substitutions per site per year (95 % HPD: 6.54×10^−4^ to 9.51×10^−4^). However, ascertainment bias occurs when the data collected do not accurately represent the population due to biased collection methods, particularly in SNV analyses. This bias can lead to incorrect conclusions about the frequency and distribution of genetic variants. Correcting for this bias provides a more accurate depiction of genetic diversity. We corrected for this bias using the equation below:

$$mu^{\prime }=pinv\times 0+\left(1-pinv\right)\times mu\;=\left(1-pinv\right)\times mu\;=\left(1-0.9983\right)\times 8.18\times 10^{-4}subst\;site-1\;year-1\;=1.32\times 10^{-6}subst\;site-1\;year-1$$

Where mu' is the clock rate corrected for ascertainment bias with the estimated proportion of invariant sites pinv=2 601 411/2 605 600. mu is the estimated clock rate. This translates to a genome-wide mutation rate of 1.32×10^−6^ mutations per year per site (Table S7), relative to genome size, implying that across the core-genome, approximately 3.4 new mutations arise each year for this subset of Clade 1.1 of *S. aureus* ST97, in concordance with previous studies [57,58].

### Further detection of ST97 MRSA after outbreak declared closed

Around 2 months after the closure of the outbreak a further ST97 MRSA from a clinical sample was detected on a routine weekly sequencing run from an infant who had not been present in the NICU at the time of the original outbreak. This caused significant concern in the IPC team regarding a possible ongoing reservoir of ST97 MRSA on the unit. Pod5 files for the isolate were cloud transferred to ESR later that day. By the next morning the isolate had been contextualized with the ST97 phylogeny, revealing that it did not cluster with the other outbreak isolates (Fig. S7). Plans for further ward screening and other control measures were withdrawn.

## Discussion

Genomics has emerged as a practical frontline tool for investigating healthcare-associated infections (HCAIs) with the capacity to conduct WGS within clinically relevant timeframes [15,59]. Such investigations often focus on tracing MRSA transmission using SNV distances as indicators [15,16, 60]. By integrating genomics with traditional hospital epidemiological methods, transmission routes for HCAIs can be effectively identified [15]. This integration can guide infection prevention strategies and optimize hospital resource allocation [18]. Other studies of *S. aureus* HCAIs have similarly demonstrated the superior resolution of WGS compared to traditional approaches, such as the conventional *S. aureus*-specific staphylococcal protein A (*spa*) typing, which often fails to detect transmission events and erroneously attributed unrelated isolates as HCAIs [61]. Recognizing these advantages, we implemented a prospective genomic surveillance system for *S. aureus* in the NICU starting in 2022 [17].

This analysis has demonstrated the rapid detection and control of an outbreak of ST97 MRSA on a NICU via prospective/proactive genomic surveillance using nanopore sequencing. The outbreak was detected when there were only two known cases, which was 13 days after collection of the first positive sample for MRSA. This early detection facilitated prompt screening of other infants on the ward, which rapidly revealed the extent of the outbreak and enabled colonized infants to be managed appropriately to prevent onwards transmission. This probably limited further transmission events to only two infants (infants 9 and 10, Fig. 1) and the outbreak was declared closed 38 days later. Because the outbreak was contained at an early stage with only a small number of infants involved, there was relatively little disruption to the overall functioning of the ward. Colonized infants could be managed within existing isolation rooms, meaning the need to close rooms or services for the purposes of cohorting colonized infants did not arise.

MRSA outbreaks in the neonatal setting are well described in the literature [9]. Many of these have reached considerable size, have persisted over long time periods and have been highly disruptive to services [11,13]. Many outbreaks occurred at a time when genomic sequencing was less available, and for others genomic sequencing was utilized but in a reactive fashion once the outbreak had already been suspected based on increased incidence [13,14]. A critical factor influencing the impact of an outbreak is speed of recognition [62]. Here we have shown that prospective/proactive genomic surveillance offers the ability to detect outbreaks at a very early stage (days compared to weeks–months [13,15]), which in this case probably limited the size of the outbreak, meaning relatively few infants were affected and the NICU could avoid some of the draconian and highly disruptive IPC interventions required to control larger outbreaks.

Nanopore sequencing offers several practical advantages over other platforms, particularly for smaller clinical laboratories where capital costs and laboratory space constraints are key considerations [17]. However, it has not typically been used as a standalone platform for investigation of outbreaks such as described in this study due to concerns regarding lower raw read accuracy [63]. Here we have shown that the addition of high-quality epidemiological information and historical sequence data help mitigate this lower accuracy, allowing clinically useful conclusions to be drawn. Strain clusters could be identified, and some isolates thought to be part of the outbreak could be excluded. The ability to rapidly exclude a subsequent ST97 case from the outbreak was of particular use (Fig. S7), because it avoided further invasive and costly IPC investigations. Whilst we could identify strain clusters through comparison with publicly available data, it is important to note that Illumina data remain essential for exploring more intricate transmission dynamics. The presence of elongated branches in nanopore-only assemblies, in contrast to shorter branches seen in short-read Illumina genomes (Fig. 3), may be attributed to the higher error rate in nanopore sequencing. These errors could introduce artificial genetic variations, potentially compromising the accuracy of the phylogenetic trees and evolutionary relationships. However, from a clinical decision-making perspective, the level of detail was sufficient for the purposes of outbreak identification, which was the primary driver of the IPC response, and fine-scale analysis of transmission dynamics was not required. Although it was not a specific aim of our analysis, another advantage of a long-read approach is the ability to better contextualize mobile antimicrobial resistance genes (e.g. on plasmids), which has particular potential advantages for hospital IPC [64].

This analysis has also demonstrated the application and potential value of a distributed sequencing approach, which may be an option for many front-line clinical microbiology laboratories that lack bioinformatics expertise but can perform the wet lab aspects of sequencing. Simple, relatively non-discriminatory sequence typing was performed in a front-line clinical laboratory. In this case, this was sufficient to identify the outbreak due to the rarity of ST97 on the NICU. However, to further define the outbreak, sequence data were transferred to ESR for more granular analysis, which contextualized the outbreak and revealed one ST97 case was unrelated to the outbreak strains. This approach proved to be extremely valuable when a further case of ST97 MRSA was detected on the unit after the closure of the outbreak and was rapidly shown to be unrelated, meaning further investigations could be avoided. Had this rapid rule-out not been possible, a persistent undetected reservoir of MRSA would have been suspected by the IPC team (e.g. a colonized staff member, or other infants who had gone undetected for an extended period). This would have probably triggered extensive screening of all infants on the unit, as well as possible screening of staff, which would have caused considerable anxiety amongst parents and staff, and generated further work for ward staff, the IPC team and the microbiology laboratory.

While our primary focus lies within healthcare settings, we observed that genomes from B371, sourced from a bovine milk tank, and H7993, obtained from a human sample, clustered together. Despite a notable distance of 148 core-genome SNVs between them, this clustering underscores intriguing connections between human and animal reservoirs of *S. aureus* (Fig. 3). Extending beyond healthcare settings, *S. aureus* impacts a range of livestock, causing complications such as mastitis in dairy-producing animals [55,65, 66], skin abscesses in rabbits [67], septicaemia and skeletal problems in broiler chickens [68], and exudative epidermitis in pigs [69]. This animal association of *S. aureus* threatens veterinary medicine, agricultural systems and food production, critical sectors for the economy in New Zealand [70]. The ST97 lineage is a prime instance of epidemic community-associated *S. aureus* in humans. The emergence of ST97 in humans can be traced back to independent jumps from cattle between 1894 and 1977 [71]. The estimated emergence of the ST97 Clade 1.1 sub-lineage around 1989 (95 % HPD: 1983–1993) suggests the MRCA probably existed post-exchange between cattle and humans, and after the introduction of methicillin in 1959 [4,72, 73], and the later emergence of resistance to methicillin [74,75].

### Conclusion

While this study focused on the detection and control of an outbreak within an NICU setting, the implications extend beyond this specific context. Prospective genomic surveillance holds promise for enhancing infection control practices across various healthcare settings, facilitating early intervention and containment of emerging threats posed by multidrug-resistant pathogens. Despite its rapid and cost-effective nature, nanopore data alone may not suffice for identifying transmissions accurately; however, by combining sequence data with epidemiological data, clinically useful conclusions can still be drawn. Continued advancements in sequencing technologies and bioinformatics tools will further bolster the capacity for timely and effective outbreak detection, ultimately safeguarding patient safety and minimizing the burden on healthcare systems.

## supplementary material

10.1099/mgen.0.001273 Uncited Supplementary Material 1.

10.1099/mgen.0.001273 Uncited Supplementary Material 2.

## Abbreviations

BAPS: Bayesian Analysis of Population Structure
BWA: Burrows-Wheeler Aligner
ESR: Institute of Environmental Science and Research
EUCAST: European Committee on Antimicrobial Susceptibility Testing
GATK: Genome Analysis Tool Kit
HCAI: healthcare-associated infection
HPC: High-Performance Compute
HPD: highest posterior density
IPC: infection prevention and control
IQR: interquartile range
LIC: Livestock Improvement Corporation
MBIE: Ministry of Business Innovation and Employment
MLST: multi-locus sequence type
MRCA: most recent common ancestor
MRSA: methicillin-resistant *Staphylococcus aureus*
MSSA: methicillin-susceptible S. aureus
NCBI: National Center for Biotechnology Information
NICU: neonatal intensive care unit
ONT: Oxford Nanopore Technologies
SCCmec: staphylococcal cassette chromosome mec
SCL: Southern Community Laboratories
SNV: single nucleotide variant
SRA: sequence read archive
ST: sequence type
WGS: whole genome sequencing
WRH: Wellington Regional Hospital
