# Supplementary material for: Rapid identification and subsequent contextualization of an outbreak of methicillin-resistant Staphylococcus aureus in a neonatal intensive care unit using nanopore sequencing
Source: Microb Genom. 2024 Jul 5;10(7):001273. doi: 10.1099/mgen.0.001273 (PMC11316549; doi:10.1099/mgen.0.001273)
Supplement: Uncited Supplementary Material 1. [file mgen-10-01273-s001.pdf]

**SUPPLEMENTARY APPENDIX**

# Rapid identification and subsequent contextualisation of an outbreak of methicillin-resistant *Staphylococcus aureus* in a neonatal intensive care unit using nanopore sequencing


## 1.1 Author names

Rhys T. White[1], Sarah Bakker[1], Megan Burton[2], M. Leticia Castro[1], Christine Couldrey[3], Kristin Dyet[1], Alexandra Eustace[1], Chad Harland[3], Samantha Hutton[2], Donia Macartney-Coxson[1], Claire Tarring[2], Charles Velasco[2], Emma M. Voss[3,4], John Williamson[4], Max Bloomfield[2,5]

## 1.2 Affiliation(s)

[1]Institute of Environmental Science and Research, Health Group, Porirua 5022, New Zealand

[2]Awanui Labs Wellington, Department of Microbiology and Molecular Pathology, Wellington 6021, New Zealand

[3]Livestock Improvement Corporation, Research and Development, Newstead 3286, New Zealand

[4]University of Otago, Department of Microbiology and Immunology, Dunedin 9016, New Zealand

[5]Te Whatu Ora/Health New Zealand, Infection Prevention and Control, Capital, Coast & Hutt Valley, Wellington 6021, New Zealand

## 1.3 Corresponding author and email address

*Corresponding author: Rhys White, Institute of Environmental Science and Research, Wellington, New Zealand; Telephone: +64-4-914-0700; E-mail: rhys.white@esr.cri.nz



## 1.4 Keywords

Genomic surveillance; outbreak detection; antibiotic resistance; phylogenetic analysis; infection control

**<u>This file includes the following:</u>**

**Supplementary Methods.**

**Supplementary Figure S1.** Major genomic elements in methicillin-resistant *Staphylococcus aureus* strain 23MR1425.

**Supplementary Figure S2.** Genomic comparisons between the I 23MR1425 chromosome and other complete *S. aureus* genomes**.**

**Supplementary Figure S3.** Maximum likelihood phylogeny of *Staphylococcus aureus* sequence type (ST)97.

**Supplementary Figure S4.** Maximum likelihood phylogeny of a subset of Clade 1.1 *Staphylococcus aureus* sequence type (ST)97.

**Supplementary Figure S5.** Single-nucleotide variant (SNV) plot.

**Supplementary Figure S6.** Maximum likelihood phylogeny of a subset of Clade 1.1 *Staphylococcus aureus sequence type* (ST)97.

**Supplementary Figure S7.** Maximum parsimony phylogeny of a subset of Clade 1.1 *Staphylococcus aureus* sequence type (ST)97.

# 2. Supplementary Methods

## 2.1 Sampling and extraction of DNA

This dataset comprises two distinct sets of *Staphylococcus aureus* samples. The first set consists of two methicillin-sensitive ST97 *S. aureus* cases collected in May 2022 and February 2023. These initial cases, represented by samples sa220609barcode87 and sa230215barcode55, were obtained from eye swabs using the standard bacterial swab technique in Amies transport medium. Subsequently, these swabs were cultured on 5% sheep blood agar at 37°C for 48 hours. For methicillin-resistant *S. aureus* (MRSA) screening, swabs were collected from various sites, including the nose, axilla, umbilicus, groin, and any open wounds. These swabs underwent culture for 18–24 hours on the CHROMagar™ MRSA (St-Denis, France) and enrichment in 7% salt broth for 18 hours, with subculture onto CHROMagar™ MRSA for a further 18–24 hours. CHROMagar MRSA is a selective and differential agar, which inhibits the growth of MSSA and most other bacteria. Mauve-coloured colonies were followed up as possible MRSA. Susceptibility to multiple other agents was determined for isolates confirmed as MRSA, using the Vitek II instrument (bioMerieux, Marcy-l'Etoile, France) and the AST-P656 card. In both groups of samples, the primary samples were promptly processed upon arrival at Awanui Laboratories Wellington.

Gram-positive DNA extraction involves suspending a 10μL loop of bacteria in 300uL of phosphate-buffered saline (PBS) and freezing at −20°C for at least 24 hours. The thawed solution is vortexed for one minute with a small quantity of 0.1mm zirconia/silica beads (dnature, Gisborne, NZ; SKU 11079101Z) and then centrifuged at ~12,000×g for 30 seconds at room temperature, with the supernatant used for sequencing without further clean-up.

## 2.2 Quality control for the nanopore sequence data

To perform taxonomic profiling and detect *S. aureus* in the raw nanopore sequence data, we used Kraken v2.1.3 [1] with default parameters and an NCBI Reference Sequence (RefSeq) database [2], Standard (https://benlangmead.github.io/aws-indexes/k2, accessed on 15 September 2023). The database contained references for archaea, bacteria, human, viruses, plasmids, and the 'UniVec core' subset of the UniVec database (a database of vector, adaptor, linker, and primer sequences). NanoStat v1.6.0 from Nanopack v1.6.0 [3] was used to perform an initial quality assessment on the raw nanopore reads. Additionally, NanoQC v0.9.4 from NanoPack was used to assess the overall quality of the sequencing data. NanoFilt v2.8.0, also from NanoPack, was used for read trimming. Initially, 50 nucleotides were trimmed from the start and end of each read to remove low-quality regions from the reads. Subsequently, NanoFilt was used again to filter out reads with a quality score below either Q10, Q12, or Q15. Any remaining reads that were less than 100 base pairs in length were also removed.

## 2.3 *de novo* assembly of the nanopore sequence read data

Filtered sequence reads were *de novo* assembled using Flye v2.9.2 [4, 5] with parameters set to: (i) estimate the genome size to 2.8 Mb; and (ii) three polishing iterations. The assemblies underwent three rounds of

additional polishing by mapping the corresponding nanopore reads to each contig using minimap2 v2.24 [6, 7], and then correcting single nucleotide variants (SNVs) and insertions and deletions (INDELs) with racon v1.4.3 [8] with parameters: '--match 8' for match score, '--mismatch -6' for mismatch score, and '--gap -8' for gap penalty. After using the nanopore reads to polish with racon, the assemblies were further refined using medaka v1.8.0 (https://github.com/nanoporetech/medaka, accessed on 15 September 2023), using the 'super accuracy' models. Subsequently, the corrected assembly was circularised using Circlator v1.5.1 [9]. A final round of polishing was completed using medaka. The assembly metrics were assessed using QUAST v5.0.2 [10].

In the absence of an available ST97 reference genome representing New Zealand *S. aureus* strains, we selected 23MR1425, a clinical isolate collected from a neonatal eye swab, as our reference genome (index case on the WRH NICU identified in June 2023). The assembly for 23MR1425 underwent five rounds of additional polishing by mapping the corresponding Illumina reads to each contig using the Burrows-Wheeler Aligner (BWA) v0.7.17 [11], and then correcting SNVs and insertions and deletions (INDELs) with Pilon v1.24 [12].

### 2.4 *In silico* genotyping of the outbreak-associated *S. aureus* genome sequence data

*In silico* multi-locus sequence typing (MLST) was done using MLST v2.9 (https://github.com/tseemann/mlst, accessed on 15 September 2023) with default settings to query the assemblies against the *Staphylococcus aureus* typing database hosted on PubMLST [13]. The spa types were identified using spaTyper v1.0.0 [14], a tool that utilised the spa typing website (http://www.spaserver.ridom.de/, accessed on 15 September 2023) developed by Ridom GmbH and maintained by SeqNet.org (http://www.SeqNet.org/, accessed on 15 September 2023). ABRicate v1.0.1 (https://github.com/tseemann/abricate, accessed on 15 September 2023) was used to screen the assemblies for acquired antibiotic resistance genes using the ARG-ANNOT [15] database (last updated 15 September 2023). SCCmec types for the 13 MRSA ST97 outbreak-associated genomes were determined using SCCmecFinder v1.2 [16].

### 2.5 Dataset curation of publicly available data

In addition to the *S. aureus* ST97 genomes sequenced in this study (Supplementary Materials, Table S1), six complete ST97 genome assemblies were downloaded from the NCBI Assembly database. Another 468 publicly available draft *S. aureus* genome assemblies were downloaded using the PathogenWatch platform (https://pathogen.watch/, accessed on 25 August 2023). Furthermore, this study included an additional 18 publicly available bovine-derived genomes [17] to this dataset, including 16 ST97 strains and one each of ST6164 and ST6162 (both clonal complex 97).

### 2.6 Illumina library construction and next-generation sequencing

As part of the Institute of Environmental Science and Research (ESR) national staphylococcal surveillance surveys, *Staphylococcus* cultures are plated on blood agar and incubated at 35°C with 5% carbon dioxide ($CO_2$) for 18 hours. Following incubation, the cultures are examined for viability and purity. A subculture of a single

colony pick is then transferred to another blood agar plate and incubated at the same conditions for 18 hours. The heat-killed cell suspensions from an overnight culture of a single colony pick were extracted using the chemagic™ 360 instrument (PerkinElmer Inc., Waltham, Massachusetts, United States). The DNA library was created using the PlexWell Library Preparation kit (seqWell™, Boston, Massachusetts, United States), and sequenced as 2×151 bp paired-end reads on the NextSeq 550 platform using V2.5 chemistry (Illumina Inc., San Diego, California, United States) at ESR (Kenepuru, Porirua, New Zealand).

For the Livestock Improvement Corporation (LIC) (Newstead, New Zealand), *S. aureus* isolates were cultured from bulk tank milk samples collected as part of a nationwide bulk tank trial for LIC Milkomics®, bulk tank milk samples undergoing animal health testing and from individual quarter foremilk samples. Ethics approval for the bulk tank milk sampling was not required, however ethics approval for the individual quarter foremilk samples was obtained from the Ruakura Animal Ethics Committee, Hamilton/Kirikiriroa, North Island/Te Ika-a-Māui, (Approval Numbers: 14240 and 15575). The bulk tank milk samples for the Milkomics® trial were collected in a 35mL pottle containing 0.1mL of bronopol preservative. Pottles were delivered to the LIC Animal Health Laboratory located in Hamilton/Kirikiriroa, North Island/Te Ika-a-Māui by LIC herd testing. The bulk tank milk samples for animal health testing were collected from the animal health laboratory and a subsample was used for bacteriology. 100µl of bulk tank milk was initially spread plated onto Esculin Sheep Blood Agar plates using a Copan spreader. These plates were then incubated at 37°C for 48 hours. The individual quarter foremilk samples were collected from selected cows based on elevated somatic cell counts. The milk samples were collected using standard aseptic technique by LIC technicians (North Island/Te Ika-a-Māui) and were taken back to the LIC Animal Health Laboratory. For South Island/Te Waka-o-Māui farms accredited veterinarians collected samples and the pottles were couriered chilled at 4°C to the LIC Animal Health Laboratory. 10µl of foremilk was spread on Esculin Sheep Blood Agar plate using a Copan spreader. The plates were incubated for 48 hours at 37°C.

Presumptive *S. aureus* isolates for bulk tank/foremilk quarter samples were identified based on the presence of incomplete or complete haemolysis zones, positive catalase reactions, and positive rabbit-plasma coagulase reactions. To ensure the purity of the positive *S. aureus* isolates, strains were sub-cultured on Esculin Sheep Blood Agar plates twice. After confirming their purity, two to three colonies from each isolate were transferred into Laboratoire de Santé Publique du Québec (LSPQ) preservation medium and stored at −20°C for future use.

Thirteen human *S. aureus* isolates were obtained from the Pathlab on nutrient agar slopes. The Pathlab identified these isolates as methicillin-susceptible *S. aureus*, isolated from the Waikato, Lakes, and Bay of Plenty/Te Moana-a-Toi regions and no identifying information was obtained, therefore no ethics approval was required. Human isolates were prepared in the same manner as the bovine isolates with storage in LSPQ media.

For genomic DNA extraction, samples were cultivated from LSPQ stocks. Genomic DNA extraction was performed using a custom BioSprint® 96 DNA kit (Qiagen), and a Kingfisher machine (ThermoFisher, New Zealand). Samples were eluted into 100µl of elution buffer.

Following extraction, DNA concentrations were assessed for all samples, including both positive and negative controls, using a Victor Nivo in a 96-well plate format using the manufacturer's protocol for the 1×dsDNA Broad Range Assay Kit (ThermoFisher, New Zealand). All isolates exhibited DNA concentrations exceeding 100ng/µL, while negative controls returned negative results. Subsequently, DNA concentrations were normalised to approximately 15ng/µL before proceeding with Illumina sequencing. The DNA was then stored at −20°C. The *S. aureus* genomes were sequenced in-house at LIC on an Illumina Novaseq 6000 using an S1 flow cell and utilising XP loading. For all 140 isolates, 150 bp paired-end Illumina sequencing reads were generated. The DNA library was created using the Illumina DNA preparation kit with Integrated DNA Technologies (IDT) for Illumina UD Indexes (Plate A/Set 1, Plate B/Set 2, Plate C/Set 3, Plate D/Set 4), and sequenced as 2×151 bp paired-end reads on the Novaseq 6000 on an S1 flow cell utilising XP loading (Illumina Inc., San Diego, California, United States) at LIC (Newstead, New Zealand).

### 2.7 Quality trimming and *de novo* assembly of the Illumina sequence read data

Raw reads were checked for quality using FastQC v0.11.9 (http://www.bioinformatics.babraham.ac.uk/projects/fastqc/, accessed on 15 September 2023). To perform taxonomic profiling and detect *S. aureus* in the raw Illumina sequence data, we used Kraken v2.1.3. Raw sequence reads were *de novo* assembled using Shovill v1.1.0 (https://github.com/tseemann/shovill, accessed on 15 September 2023), which utilises: Seqtk v1.3-r106 (https://github.com/lh3/seqtk, accessed on 15 September 2023); Trimmomatic v0.36 [18]; Lighter v1.1.2 [19]; FLASH v1.2.11 [20]; SKESA v2.4.0 [21, 22]; Samclip v0.4.0 (https://github.com/tseemann/samclip, accessed on 15 September 2023); SAMtools v1.16.1 [23], BWA v0.7.17 [11]; and Pilon v1.24 [12]. Shovill was used with parameters set to: (i) estimate the genome size to 2.8 Mb; (ii) remove contiguous sequences (contigs) with a sequence coverage below 20-fold; and (iii) enable single-cell mode. Assembly metrics were assessed using QUAST v5.0.2 [24]. The quality metrics were assessed and are outlined in the Supplementary Materials (Table S8).

### 2.8 Detecting recombination in the subset of Clade 1.1 ST97 from New Zealand

A core-genome alignment was generated from 97 *S. aureus* ST97 genome assemblies using Parsnp v1.7.4 [25] with the 23MR1425 chromosome serving as the reference to call SNVs (index ST97 MRSA case, date sample received June 2023). Resulting SNV alignments were used to reconstruct phylogenies. Maximum likelihood phylogenetic trees were reconstructed using RaxML v8.2.12 [26] (GTR-GAMMA correction) by optimising 20 distinct, randomised maximum parsimony trees before adding 1,000 bootstrap replicates. Putative recombinogenic regions were detected and a recombination-corrected phylogeny was built with ClonalFrameML v.1.13 [27]. The resulting phylogenetic trees were visualised using FigTree v1.4.4 (http://tree.bio.ed.ac.uk/software/figtree/, accessed on 18 March 2024).

# 3. Supplementary Figures

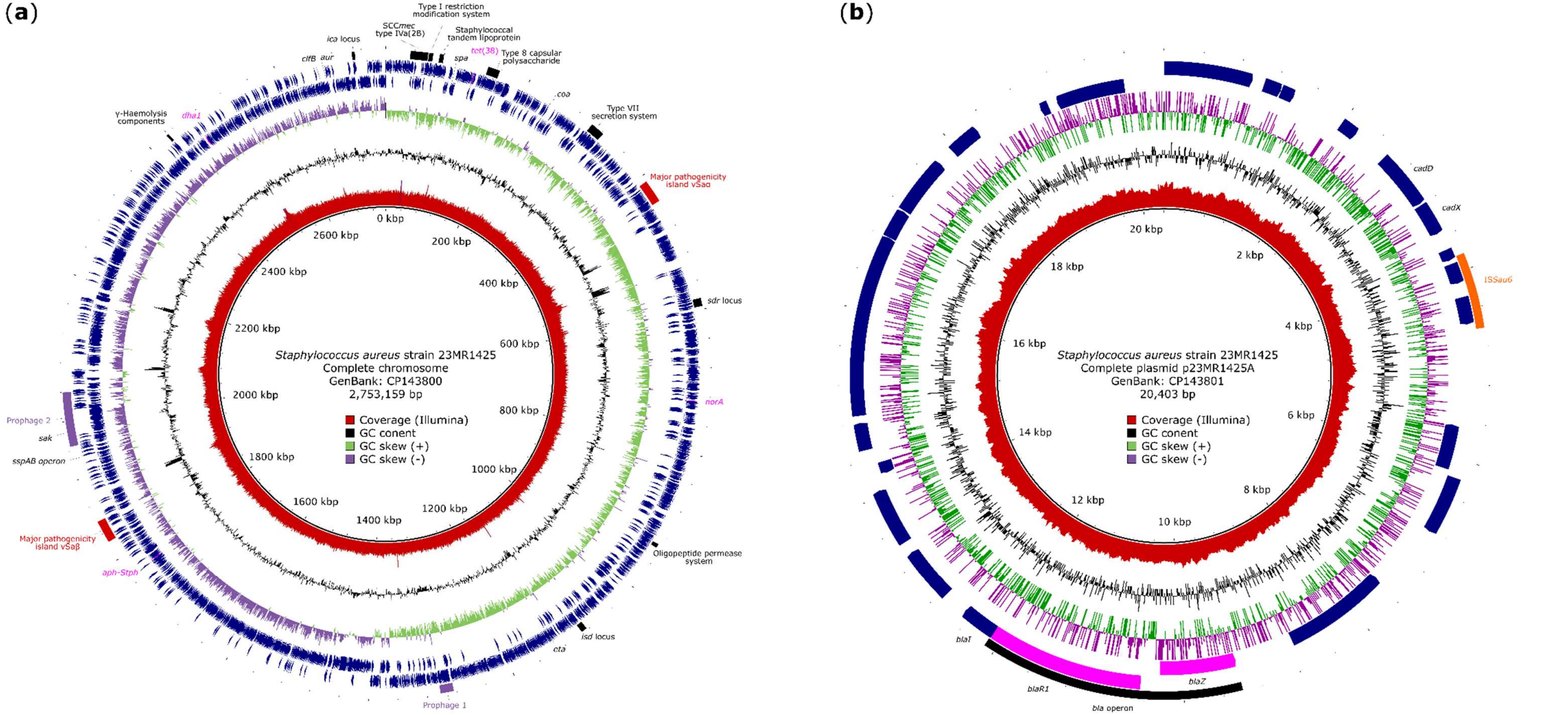


**Supplementary Figure S1. Major genomic elements in methicillin-resistant *Staphylococcus aureus* strain 23MR1425.** Circular representation of the *S. aureus* 23MR1425 chromosome (**a**) and plasmid (**b**). The four innermost circles represent chromosome coordinates relative to 23MR1425, genome coverage of mapped Illumina reads (graph maximum value 250× in a and 1250× in b), GC content, and GC skew. Rings 5 and 6 represent the 23MR1425 coding sequences encoded on the reverse and forward strand, respectively. The outermost ring describes the positions of regions of interest (black), genomic islands (red), and prophages (purple). Antibiotic resistance genes are coloured pink. Image created using BRIG [28].

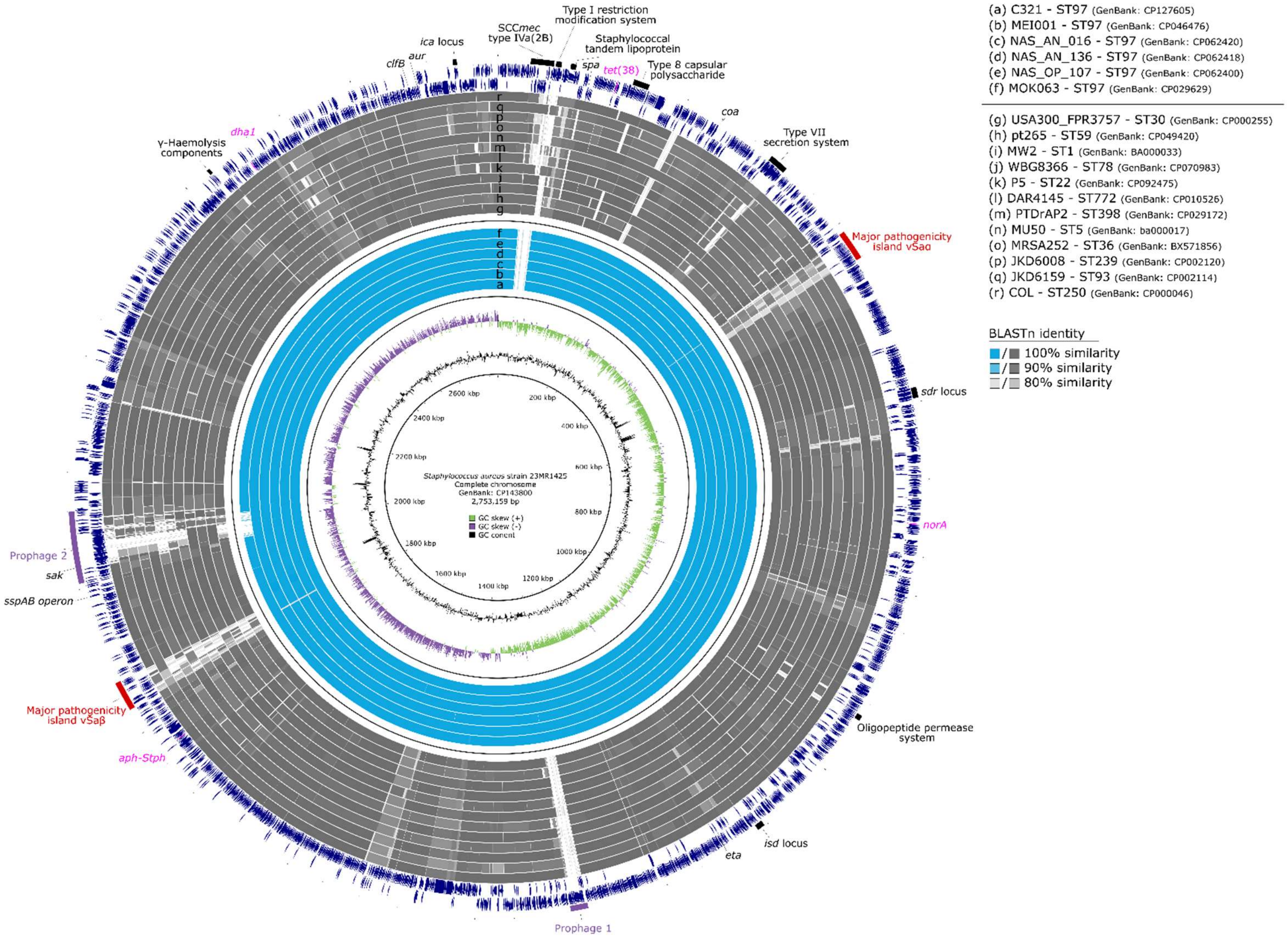


**Supplementary Figure S2. Genomic comparisons between the *Staphylococcus aureus* 23MR1425 chromosome and other complete *S. aureus* genomes.** Circular representation of the *S. aureus* 23MR1425 chromosome. The three innermost circles represent chromosome coordinates relative to 23MR1425, GC content, and GC skew. The degree of coloured shading indicates nucleotide identity between 23MR1425 and each *S. aureus* chromosome (rings labelled a to r). Key is ordered with the innermost ring at the top, descending to the outermost ring. Ligh blue rings correspond to other *S. aureus* sequence type ST97 genomes, as shown in the legend. The outermost ring describes the positions of regions of interest (black), genomic islands (red), and prophages (purple). Image created using BRIG [28].

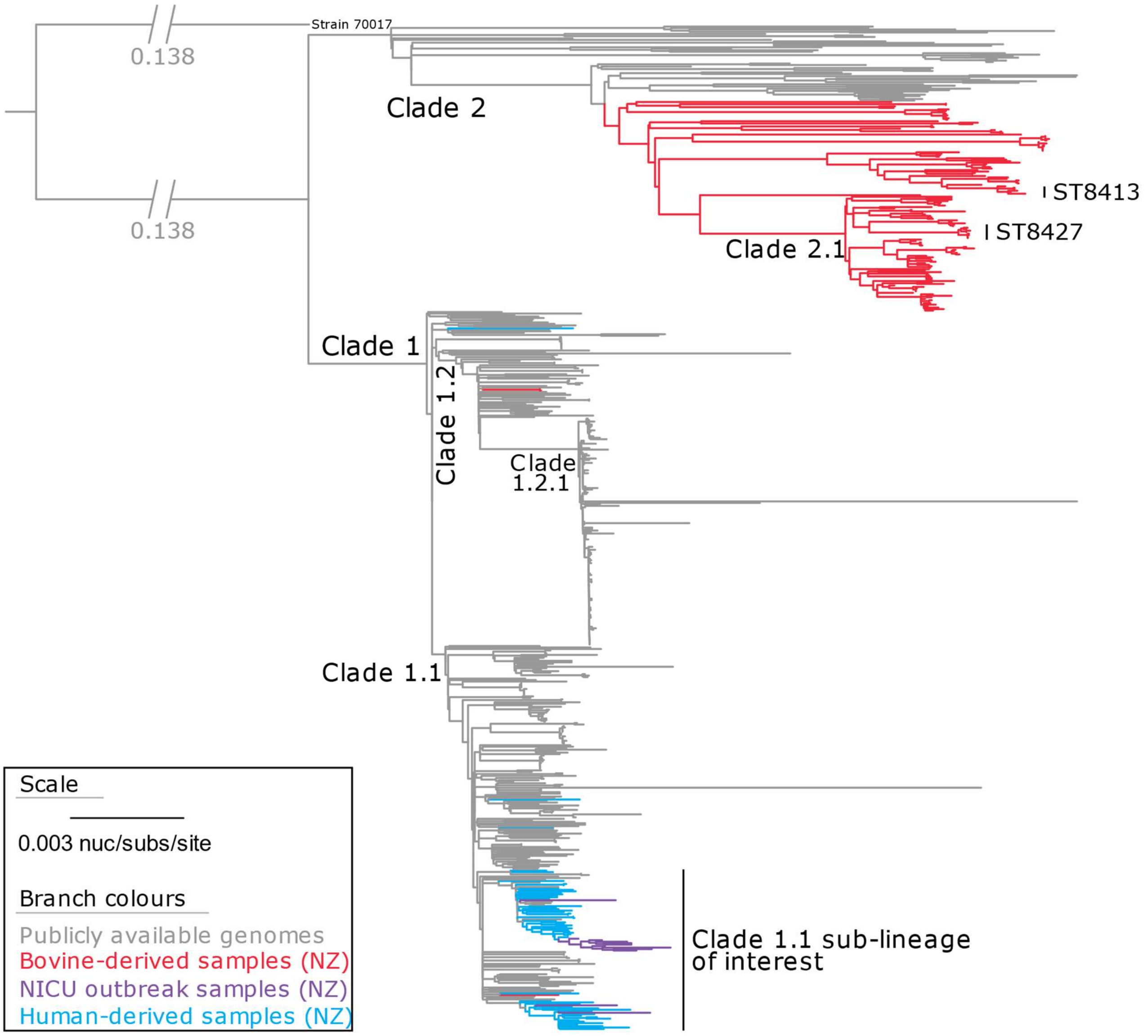


**Supplementary Figure S3. Maximum likelihood phylogeny of *Staphylococcus aureus* sequence type (ST)97**. The phylogeny was inferred from 36,196 core-genome single-nucleotide variants (SNVs) from 680 assembled genomes. SNVs were derived from a core-genome alignment of 2,072,556 bp and are called against the chromosome of sample 23MR1425.

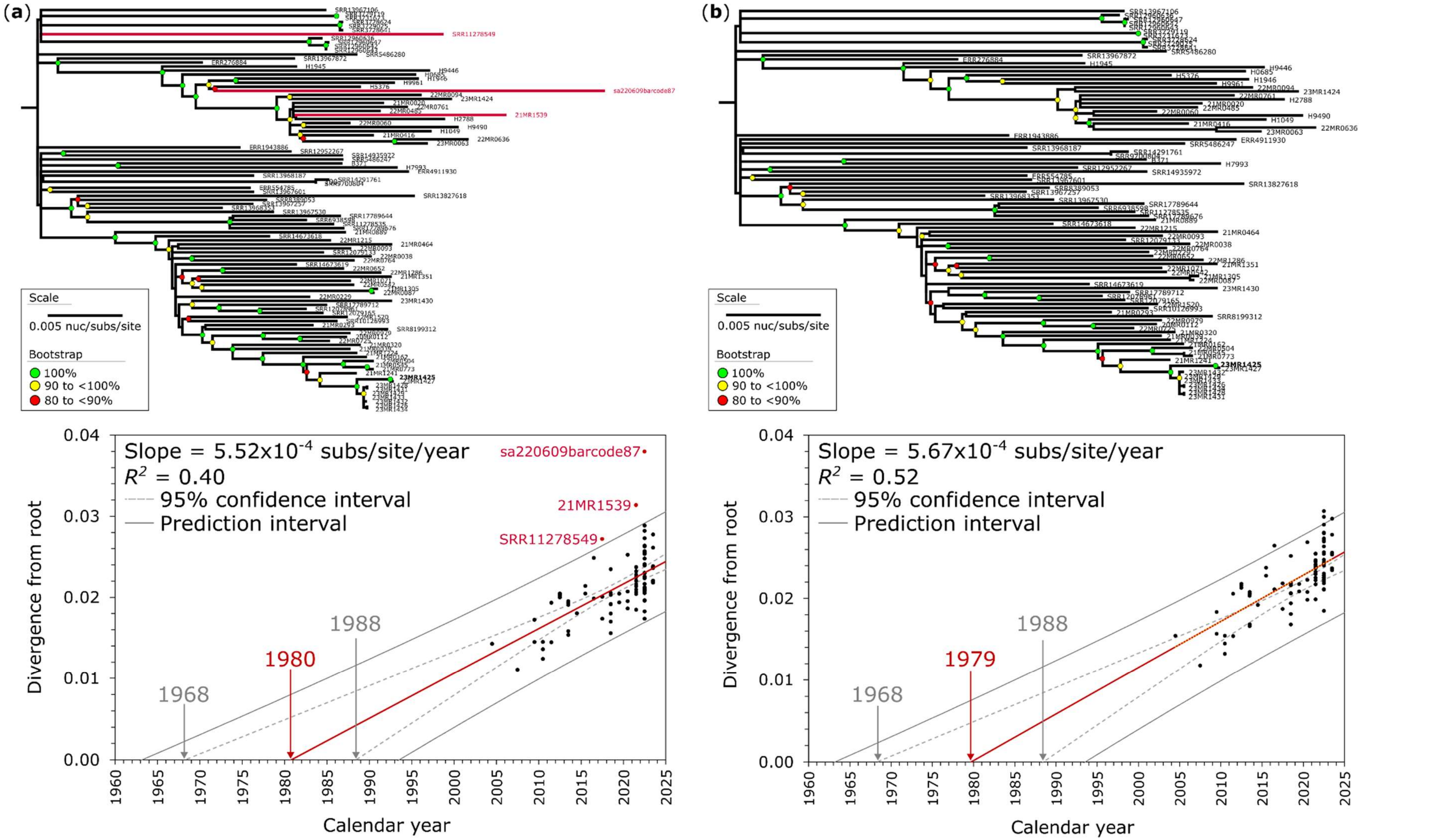


**Supplementary Figure S4. Maximum likelihood phylogeny of a subset of Clade 1.1 *Staphylococcus aureus* sequence type (ST)97**. (a) The phylogeny was inferred from 4,457 core-genome single-nucleotide variants (SNVs) from 100 genomes. SNVs were derived from a core-genome alignment of ~2,601,900 bp and are called against the chromosome of sample 23MR1425. (b) The phylogeny was inferred from 4,189 core-genome SNVs from 97 genomes. SNVs were derived from a core-genome alignment of ~2,605,600 bp and were called against the chromosome of sample 23MR1425. The genome for sample sa220609barcode87 represents nanopore only sequence data. Both phylogenetic trees are rooted according to the ERR4911723 outgroup. Bootstrap values >80% (1,000 replicates) are shown.

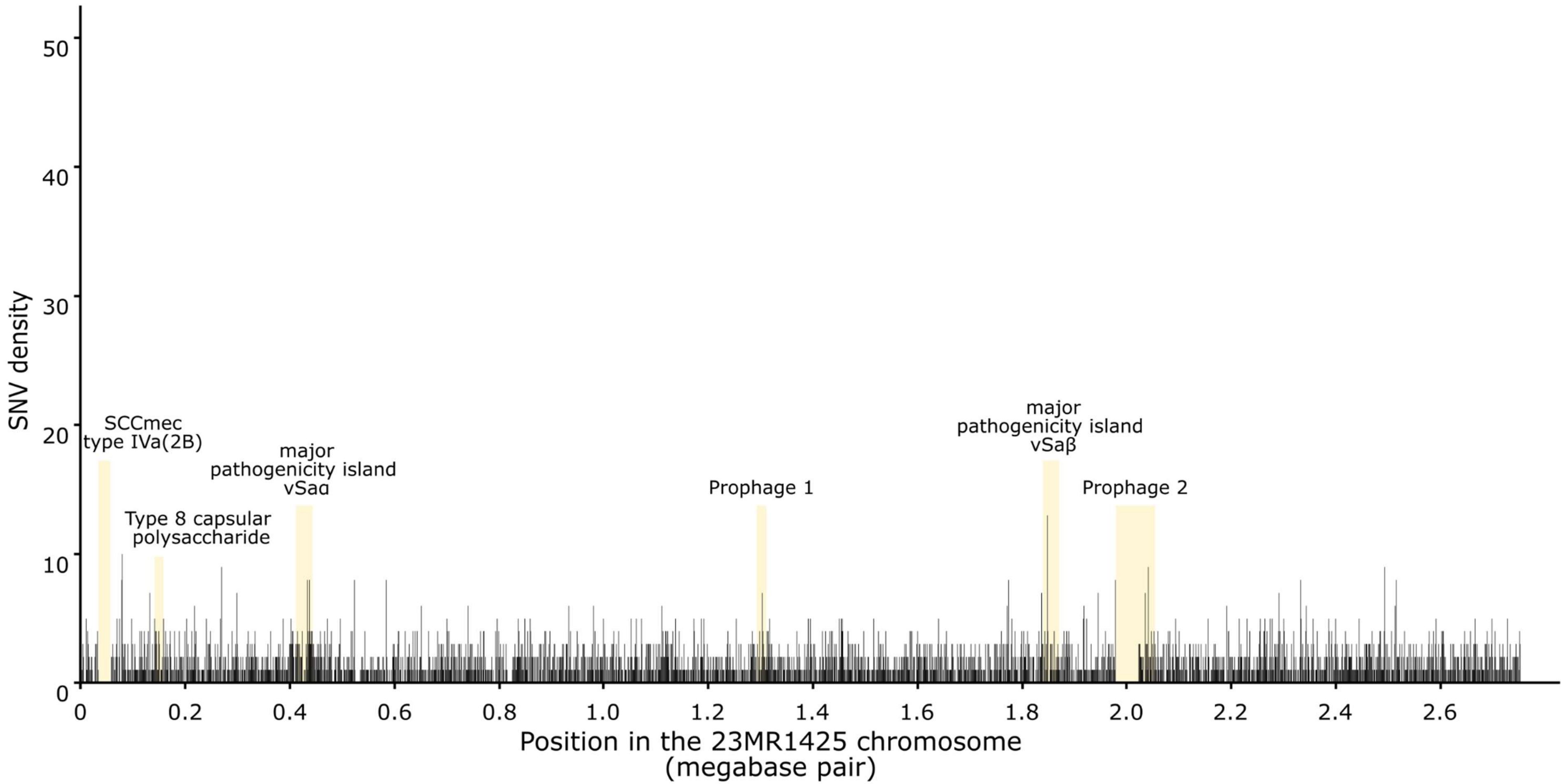


**Supplementary Figure S5. Single-nucleotide variant (SNV) plot.** This plot is based on the alignment of 4,189 core-genome SNV from a total of 97 ST97 genomes and represents the number of SNVs in a 1,000 bp sliding window relative to the 23MR1425 reference chromosome**.**

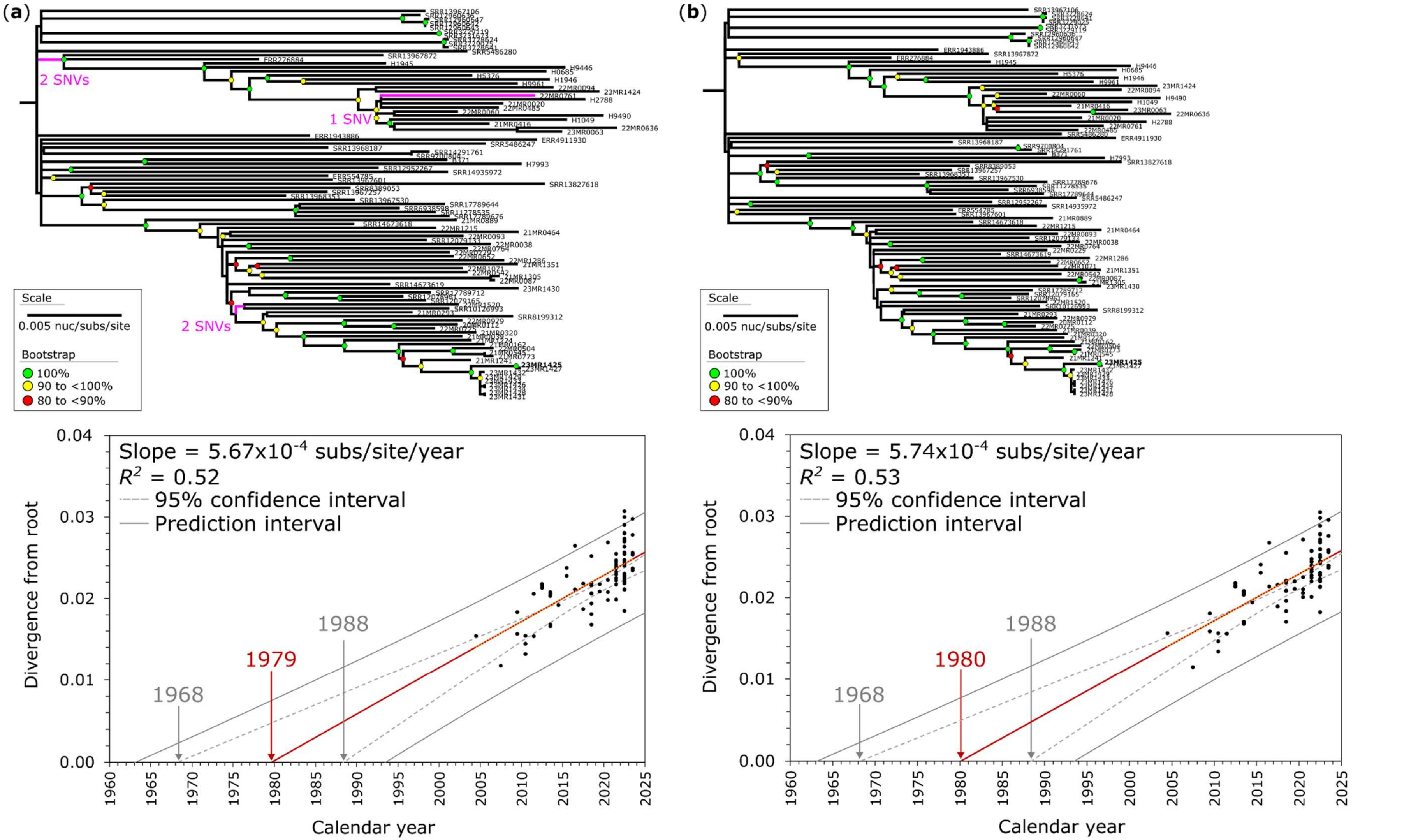


**Supplementary Figure S6. Maximum likelihood phylogeny of a subset of Clade 1.1 *Staphylococcus aureus* sequence type (ST)97**. (a) The phylogeny was inferred from 4,189 core-genome single-nucleotide variants (SNVs) from 97 genomes, prior to additional recombination filtering. Pink branches indicate SNVs located within regions of recombination. (b) The phylogeny was inferred from 4,184 core-genome SNVs outside of regions of recombination from 97 genomes. In both trees, SNVs were derived from a core-genome alignment of ~2,605,600 bp and were called against the chromosome of sample 23MR1425. Both phylogenetic trees are rooted according to the ERR4911723 outgroup. Bootstrap values >80% (1,000 replicates) are shown.

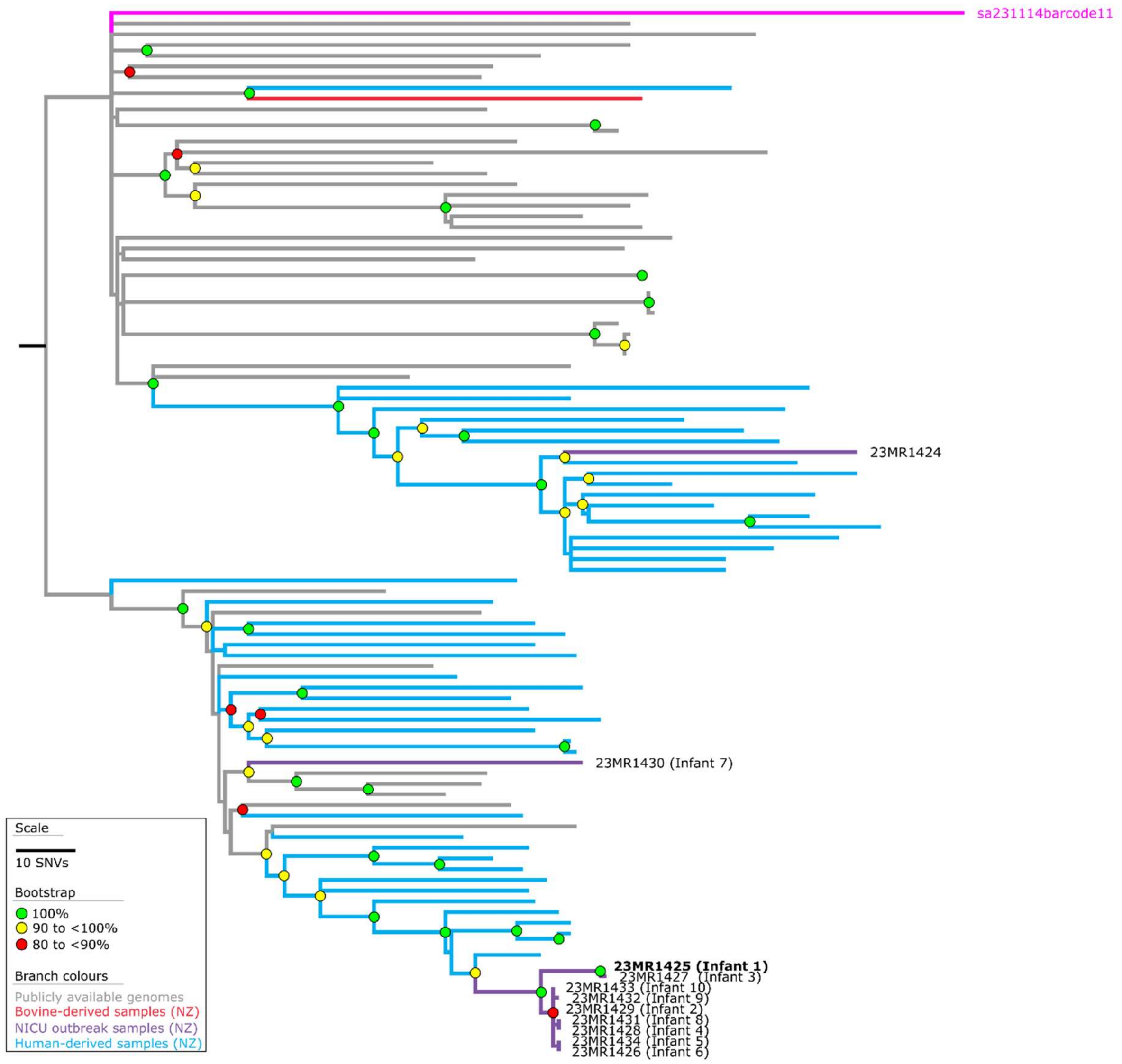


**Supplementary Figure S7. Maximum parsimony phylogeny of a subset of Clade 1.1 *Staphylococcus aureus* sequence type (ST)97**. The phylogeny was inferred from 4,285 core-genome single-nucleotide variants (SNVs) from 98 genomes. SNVs were derived from a core-genome alignment of ~2,592,800 bp and are called against the chromosome of sample 23MR1425. The genome for sample sa221114barcode11 represents nanopore only sequence data. The consistency index for the tree is 0.99. The phylogenetic tree is rooted according to the ERR4911723 outgroup. Bootstrap values >80% (1,000 replicates) are shown. Pink branch and taxa represents another ST97 genome found approximately two months after the closure of the outbreak.

## 4. References


1. **Wood DE, Salzberg SL**. Kraken: ultrafast metagenomic sequence classification using exact alignments. *Genome Biology* 2014;15:R46 doi: 10.1186/gb-2014-15-3-r46

2. **Sayers EW, Barrett T, Benson DA, Bryant SH, Canese K, Chetvernin V, *et al***. Database resources of the National Center for Biotechnology Information. *Nucleic Acids Research* 2009;37:3124 doi: 10.1093/nar/gkn741

3. **De Coster W, D'hert S, Schultz DT, Cruts M, Van Broeckhoven C**. NanoPack: visualizing and processing long-read sequencing data. *Bioinformatics* 2018;34:2666-2669 doi: 10.1093/bioinformatics/bty149

4. **Lin Y, Yuan J, Kolmogorov M, Shen MW, Chaisson M, Pevzner PA**. Assembly of long error-prone reads using de Bruijn graphs. *Proceedings of the National Academy of Sciences of the United States of America* 2016;113:E8396-E8405 doi: 10.1073/pnas.1604560113

5. **Kolmogorov M, Yuan J, Lin Y, Pevzner PA**. Assembly of long, error-prone reads using repeat graphs. *Nature Biotechnology* 2019;37:540-546 doi: 10.1038/s41587-019-0072-8

6. **Li H**. Minimap2: pairwise alignment for nucleotide sequences. *Bioinformatics* 2018;34:3094-3100 doi: 10.1093/bioinformatics/bty191

7. **Li H**. New strategies to improve minimap2 alignment accuracy. *Bioinformatics* 2021;37:4572-4574 doi: 10.1093/bioinformatics/btab705

8. **Vaser R, Sović I, Nagarajan N, Šikić M**. Fast and accurate de novo genome assembly from long uncorrected reads. *Genome Research* 2017;27:737-746 doi: 10.1101/gr.214270.116

9. **Hunt M, Silva ND, Otto TD, Parkhill J, Keane JA, Harris SR**. Circlator: automated circularization of genome assemblies using long sequencing reads. *Genome Biology* 2015;16:294 doi: 10.1186/s13059-015-0849-0

10. **Gurevich A, Saveliev V, Vyahhi N, Tesler G**. QUAST: quality assessment tool for genome assemblies. *Bioinformatics* 2013;29:1072-1075 doi: 10.1093/bioinformatics/btt086

11. **Li H, Durbin R**. Fast and accurate short read alignment with Burrows-Wheeler transform. *Bioinformatics* 2009;25:1754-1760 doi: 10.1093/bioinformatics/btp324

12. **Walker BJ, Abeel T, Shea T, Priest M, Abouelliel A, Sakthikumar S, *et al***. Pilon: an integrated tool for comprehensive microbial variant detection and genome assembly improvement. *PLOS ONE* 2014;9:e112963 doi: 10.1371/journal.pone.0112963

13. **Jolley KA, Bray JE, Maiden MC**. Open-access bacterial population genomics: BIGSdb software, the PubMLST.org website and their applications. *Wellcome Open Research*, 2018;3:124 doi: 10.12688/wellcomeopenres.14826.1

14. **Harmsen D, Claus H, Witte W, Rothganger J, Claus H, Turnwald D, Vogel U**. Typing of methicillin-resistant *Staphylococcus aureus* in a university hospital setting by using novel software for *spa* repeat determination and database management. *Journal of Clinical Microbiology* 2003;41:5442-5448 doi: 10.1128/jcm.41.12.5442-5448.2003

15. **Gupta SK, Padmanabhan BR, Diene SM, Lopez-Rojas R, Kempf M, Landraud L, *et al*.** ARG-ANNOT, a new bioinformatic tool to discover antibiotic resistance genes in bacterial genomes. *Antimicrobial Agents and Chemotherapy* 2014;58:212-220 doi: 10.1128/AAC.01310-13

16. **Kaya H, Hasman H, Larsen J, Stegger M, Johannesen TB, Allesøe RL, *et al*.** SCCmecFinder, a web-based tool for typing of staphylococcal cassette chromosome *mec* in *Staphylococcus aureus* using whole-genome sequence data. *mSphere* 2018;3:e00612-e00617. doi: 10.1128/msphere.00612-17

17. **Nesaraj J, Grinberg A, Laven R, Biggs P**. Genomic epidemiology of bovine mastitis-causing *Staphylococcus aureus* in New Zealand. *Veterinary Microbiology* 2023;282:109750 doi: 10.1016/j.vetmic.2023.109750

18. **Bolger AM, Lohse M, Usadel B**. Trimmomatic: a flexible trimmer for Illumina sequence data. *Bioinformatics* 2014;30:2114-2120 doi: 10.1093/bioinformatics/btu170

19. **Song L, Florea L, Langmead B**. Lighter: fast and memory-efficient sequencing error correction without counting. *Genome Biology* 2014;15:509 doi: 10.1186/s13059-014-0509-9

20. **Magoc T, Salzberg SL**. FLASH: fast length adjustment of short reads to improve genome assemblies. *Bioinformatics* 2011;27:2957-2963 doi: 10.1093/bioinformatics/btr507

21. **Souvorov A, Agarwala R**. SAUTE: sequence assembly using target enrichment. *BMC Bioinformatics* 2021;22:375 doi: 10.1186/s12859-021-04174-9

22. **Souvorov A, Agarwala R, Lipman DJ**. SKESA: strategic k-mer extension for scrupulous assemblies. *Genome Biology* 2018;19:153 doi: 10.1186/s13059-018-1540-z

23. **Li H, Handsaker B, Wysoker A, Fennell T, Ruan J, Homer N, *et al*.** The sequence alignment/map format and SAMtools. *Bioinformatics* 2009;25:2078-2079 doi: 10.1093/bioinformatics/btp352

24. **Gurevich A, Saveliev V, Vyahhi N, Tesler G**. QUAST: quality assessment tool for genome assemblies. *Bioinformatics* 2013;29:1072-1075 doi: 10.1093/bioinformatics/btt086

25. **Treangen TJ, Ondov BD, Koren S, Phillippy AM.** The Harvest suite for rapid core-genome alignment and visualization of thousands of intraspecific microbial genomes. *Genome Biology* 2014;15:524 doi: 10.1093/molbev/msh118

26. **Stamatakis A**. RAxML version 8: a tool for phylogenetic analysis and post-analysis of large phylogenies. *Bioinformatics* 2014;30:1312-1313 doi: 10.1093/bioinformatics/btu033

27. **Didelot X, Wilson DJ**. ClonalFrameML: efficient inference of recombination in whole bacterial genomes. *PLoS Computational Biology* 2015;11:e1004041 doi: 10.1371/journal.pcbi.1004041

28. **Alikhan NF, Petty NK, Ben Zakour NL, Beatson SA**. BLAST Ring Image Generator (BRIG): simple prokaryote genome comparisons. *BMC Genomics* 2011;12:402 doi: 10.1186/1471-2164-12-402
